# Association between Preoperative Hand Grip Strength and Postoperative Delirium after Cardiovascular Surgery: A Retrospective Study

**DOI:** 10.3390/jcm12072705

**Published:** 2023-04-04

**Authors:** Taichi Kotani, Mitsuru Ida, Satoki Inoue, Yusuke Naito, Masahiko Kawaguchi

**Affiliations:** 1 Department of Anesthesiology, Nara Medical University, Kashihara 634-8522, Japan; tk10830715@gmail.com (T.K.); schneider.yusuke@gmail.com (Y.N.); drjkawa@gmail.com (M.K.); 2 Department of Anesthesiology, Fukushima Medical University, Fukushima 960-1295, Japan; seninoue@fmu.ac.jp

**Keywords:** cardiovascular surgical procedures, delirium, hand strength, postoperative period, postoperative cognitive complications

## Abstract

The association of frailty with postoperative delirium has not been fully investigated in patients undergoing cardiovascular surgery. Therefore, this study aimed to investigate whether preoperative hand grip strength is associated with postoperative delirium. This retrospective study included patients aged >65 years who had undergone elective cardiovascular surgery using cardiopulmonary bypass at a Japanese university hospital between April 2020 and February 2022. We defined low hand grip strength as hand grip values of <275 *n* and <177 *n* for men and women, respectively. Postoperative delirium was assessed using the confusion assessment method during patients’ intensive care unit stay. The odds ratio of low hand grip strength for postoperative delirium was estimated using multiple logistic analysis, which was adjusted for prominent clinical factors. Ninety-five patients with a median age of 74 years were included in the final analysis, and 31.5% of them had low hand grip strength. Postoperative delirium occurred in 37% of patients, and the odds ratio of low preoperative hand grip strength for postoperative delirium was 4.58 (95% confidence interval: 1.57–13.2). Thirty-seven patients experienced postoperative delirium after cardiovascular surgery using cardiopulmonary bypass, and low preoperative hand grip strength was positively associated with its occurrence.

## 1. Introduction

Delirium in the intensive care unit (ICU) is an independent factor associated with increased mortality [1]; thus, prevention of delirium is important in postoperative management. 

Recently, biological age-related features, such as frailty, have received attention because of their negative impact on the development of postoperative delirium [2,3]. Although frailty has been defined using various definitions, the classic definition introduced by Fried et al. includes the following five items: weight loss, exhaustion, low activity, slow walk, and low hand grip strength [4]. In daily clinical practice, it is time- and manpower-consuming to assess the five aforementioned items. Conversely, hand grip strength can be measured simply and quickly, and some previous studies including surgical and non-surgical hospitalized patients showed that low hand grip strength was associated with the development of delirium [5,6]; however, these associations in patients undergoing cardiac surgery have been poorly documented.

Therefore, this study aimed to investigate the association between low preoperative hand grip strength values and postoperative delirium in patients undergoing cardiovascular surgery. We hypothesized that the onset of delirium would prolong ICU stays and increase the number of CAM-ICU evaluations.

## 2. Materials and Methods

### 2.1. Ethical Approval

This retrospective study was approved by the Institutional Review Board of Nara Medical University (approval number: 2958). The requirement for informed consent was waived owing to the retrospective nature of the study. This analyses adhered to the guidelines of Strengthening the Reporting of Observational Studies in Epidemiology.

### 2.2. Inclusion and Exclusion Criteria

This study included patients aged >65 years who had undergone elective cardiovascular surgery using cardiopulmonary bypass at our hospital between April 2020 and February 2022. The exclusion criteria were emergency operation, preoperatively diagnosed dementia, psychiatric conditions requiring treatment, impaired hand grip strength measurement due to upper extremity disability or unstable angina, off-pump coronary artery bypass graft (CABG), intraoperative death, or death during the ICU stay. 

### 2.3. Data Collection

We retrospectively collected preoperative data from eligible patients. These included the JapanSCORE2, which allows the prediction of mortality and major complications at 30 days after coronary, valve, and aortic surgery and allows for the estimation of surgical risk, including postoperative complications and death [7].

### 2.4. Patient Management

Because of the retrospective study design, all anesthetic management, including fluid administration and management of blood pressure, was dependent on the anesthesiologist’s discretion. The use of cardiopulmonary bypass for CABG was determined in advance by the surgeons. Cardiovascular surgical care for patients requiring cardiopulmonary bypass was performed in accordance with the institutional protocol. All patients were transferred to the ICU postoperatively, and postoperative care was provided by surgeons.

### 2.5. Measurement of Hand Grip Strength

In our hospital, handgrip strength is routinely measured in all patients > 65-year-old as a daily practice. We used the maximum value of dominant hand grip strength, which was measured three times using a Smedley-type digital hand dynamometer (TK5401, Takei Scientific Instruments Co., Niigata Japan). The reference values of low hand grip strength in the revised Japanese version of the Cardiovascular Health Study criteria (revised J-CHS criteria) are <275 *n* (28 kgf) and <177 *n* (18 kgf) for men and women, respectively [8]; thus, we defined patients with hand grip strength below the reference values (men <275 *n*, women <177 *n*) as those with decreased hand grip strength.

### 2.6. Diagnosis of Postoperative Delirium

We assessed delirium three times a day until ICU discharge using the confusion assessment method in the ICU (CAM-ICU), following the assessment of the Richmond Agitation-Sedation Scale (RASS) score as our routine clinical practice [9]. If the patient’s conscious state was scored as −4 or −5 by the RASS [10], we deferred evaluation of their delirium and re-evaluated them 8 h later. We diagnosed delirium as at least one positive result during the ICU stay.

### 2.7. Outcomes

The primary outcomes of this study were the incidence of postoperative delirium and its association with low handgrip strength.

### 2.8. Statistical Analysis

Continuous variables and categorical data are presented as median [1st Interquartile range, 3rd Interquartile range] and cases (%), respectively. Univariate analysis was performed using the chi-square test, Fisher exact test, or unpaired *t*-test, as appropriate, to compare preoperative factors between groups with and without postoperative delirium. The odds ratios of low preoperative hand grip strength for postoperative delirium were estimated using logistic regression analysis with and without adjusting for clinically relevant factors, including age, the JapanSCORE2, intraoperative cardiopulmonary bypass time, and intraoperative blood loss [2,11]. Statistical analyses were performed using SPSS (version 25.0; IBM Corp., Armonk, NY, USA). Statistical significance was set at *p* < 0.05.

## 3. Results

**Figure 1:**
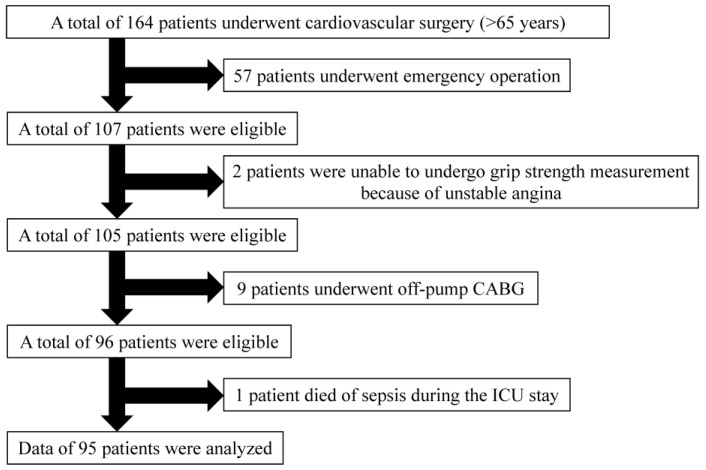
Patient flowchart. CABG: coronary artery bypass grafting, ICU: intensive care unit.

Ninety-five patients were included in this analysis, including 60 men and 35 women (Figure 1). Of these, 37% experienced postoperative delirium. As shown in Table 1, univariate analysis revealed that patients with postoperative delirium had a lower estimated glomerular filtration rate (*p* = 0.03), lower hand grip strength (men <275 *n*, women <177 *n*) (*p* = 0.002), and higher JapanSCORE2 (*p* = 0.003) than those without postoperative delirium. Table 2 shows the intraoperative and postoperative data of the patients with and without postoperative delirium. There were statistically significant differences in the duration of the ICU stay (*p* < 0.001) and number of CAM-ICU evaluations (*p* < 0.001) between patients with and without postoperative delirium. Multiple logistic analysis revealed that the odds ratio of low hand grip strength for postoperative delirium was 4.58 (95% confidence interval: 1.57–13.2) (Table 3).

In Model 1, only handgrip strength was adjusted (univariate analysis). In Model 2, hand grip strength, age, the JapanSCORE2, intraoperative cardiopulmonary bypass time, and intraoperative blood loss were adjusted (multiple analyses).

Areas under the receiver operating characteristic curve were 0.65 (95% confidence interval: 0.53–0.77) and 0.78 (95% confidence interval: 0.68–0.88) in models 1 and 2, respectively.

## 4. Discussion

The incidence of postoperative delirium after cardiovascular surgery using cardiopulmonary bypass in patients >65 years of age at our hospital was 37% (35/95 patients), and low preoperative hand grip strength was associated with its occurrence. The measurement of hand grip strength is easy to introduce in clinical practice; however, no definite grip strength values have been associated with postoperative delirium. Our study was able to demonstrate an association between low hand grip strength and postoperative delirium by using the revised J-CHS criteria reference values.

A Japanese prospective cohort study showed that low hand grip strength in midlife and late life is an important factor regarding the development of dementia and its subtypes, such as Alzheimer’s disease and vascular dementia. [11]. Additionally, hand grip strength values have been associated with cognitive function in the elderly population [12], and patients with cognitive dysfunction have an increased development of postoperative delirium [13]. Our finding that low preoperative hand grip strength was associated with postoperative delirium is in line with these previous findings. Moreover, another our study found that the exercise capacity of five or more metabolic equivalents (METs) in male patients with heart failure was equivalent to approximately 345 *n* of hand grip strength [14]. Hand grip strength, especially in patients undergoing cardiovascular surgery, may be an indirect indicator of METs, namely exercise tolerance. Taken together, these studies suggest that patients with higher hand grip strength values exercise habitually, and their hand grip strength may contribute to improving not only musculoskeletal function but also cardiovascular function. Furthermore, results of the 6 min walk test have been reported to predict postoperative delirium after transcatheter aortic valve replacement [15], which also suggests that preoperative exercise habits are associated with postoperative delirium. 

On the other hand, a recent cohort study concluded that grip strength did not contribute to discriminating the development of postoperative delirium. They used a 30-item-long Frailty Index (FI) and reported that they were associated with the occurrence of the postoperative delirium [16]. However, they have subsequently reported that a study involving a 32-item-long FI, which added grip strength and the timed-up-and-go-test, a specific measure of muscle strength, did not have a statistically stronger association with the occurrence of postoperative delirium compared to the 30-item study. They did not address the association between postoperative delirium and grip strength values alone despite comparing 30-items FI to 32-items. While this study confirms that FI is useful, 30 FI item studies have a rather large number of items. Our study used only grip strength, and we believe that a simpler method could have clarified the association with postoperative delirium.

We have not retrospectively analyzed whether hypoxia, microembolization, prolonged hypotension, or other conditions could be associated with postoperative delirium. These factors are very important risk factors for the occurrence of postoperative delirium because it is a result of neuroinflammation [17]. Unfortunately, we cannot intervene preoperatively to address these intraoperative factors. We designed this study to find preoperative factors that are associated with postoperative delirium and that can be easily measured.

Preventing delirium is an important aspect of postoperative management because delirium in the ICU is an independent factor that increases mortality [1]. Several programs have been proposed to prevent delirium after cardiovascular surgery [18], but none have focused on preoperative handgrip strength values. Our study shows that we must be aware that patients with low preoperative hand grip strength are more likely to develop postoperative delirium than those without such a weakness. Unfortunately, it has been reported that it is difficult to significantly strengthen hand grip strength using traditional resistance training [19]. Although it is difficult to improve hand grip strength, we may be able to prevent postoperative delirium by encouraging patients to exercise habitually.

If preoperative delirium can be easily predicted, it would have several advantages. First, the preoperative detection of high-risk patients may assist in correctly diagnosing postoperative delirium. Despite the high prevalence of postoperative delirium due to advanced age, impaired vision, or hypoactive subtype delirium, health care providers may recognize symptoms in only 20–50% of patients who develop delirium [20,21]. A prospective observational study showed that the risk of mortality, nosocomial infections, and pneumonia is increased in patients with delayed treatment of delirium compared to those with early detection and intervention for delirium [22]. Thus, efforts to reduce the number of missed diagnoses of delirium are important. Second, preoperative prediction may assist patients and their families in deciding whether to undergo cardiovascular surgery. Symptoms due to delirium, such as restlessness and aggressive behavior, can have potentially harmful effects on their families as well as patients. Even if patients have hypoactive subtype delirium, these symptoms affect their family just as much [23]. It may also distress their families that postoperative delirium significantly increases the duration of hospital stays and discharge to a nursing home [24]. If we could easily predict the occurrence of delirium before elective cardiovascular surgery, it may help patients and their families who are unsure whether or not to undergo cardiovascular surgery to decide to do so.

This study had some limitations. First, the number of factors in the multiple analyses was limited owing to the limited sample size. Second, we did not measure cognitive function before the operation; however, we consider that there were few patients with severe cognitive dysfunction since they were able to understand the three hand grip strength measurements. Third, the generalizability of our findings may be limited because this was a retrospective study conducted at a single hospital.

In conclusion, the incidence of postoperative delirium in patients aged >65 years after cardiovascular surgery using cardiopulmonary bypass was 37% and low preoperative hand grip strength was associated with its occurrence. Our findings may contribute to the early diagnosis of postoperative delirium in patients with low hand grip strength.

## Figures and Tables

**Table 1:** Demographic characteristics of the patients with and without postoperative delirium.

	Delirium (−) *n* = 60	Delirium (+) *n* = 35	*p*-Value
Age (years)	74 [71.7, 78]	74 [71, 77.5]	0.84
Sex (male)	44 (73)	20 (57)	0.10
Body mass index (kg/m^2^)	22.4 [20.7, 25.2]	24.3 [20.8, 26.9]	0.08
Comorbidity			
Cerebrovascular disease	11 (18)	8 (23)	0.60
Hypertension	49 (82)	25 (71)	0.25
Atrial fibrillation	8 (13)	2 (6)	0.21
Diabetes mellitus	22 (37)	13 (37)	0.96
Laboratory data			
Serum albumin level (g/dL)	4.3 [4.0, 4.5]	4.1 [3.9, 4.6]	0.65
eGFR (mL/min/1.73 m^2^)	56.0 [42.1, 68.8]	43.8 [8.7, 61]	0.03
Ejection fraction (%)	63.5 [56.2, 68]	60 [47, 70]	0.56
Hand grip strength (*n*)	304 [246, 339]	223 [174, 285]	0.001
Low hand grip strength (men < 275 *n*, women < 177 *n*)	12 (20)	18 (51)	0.002
JapanSCORE2	2.1 [1.5, 4.4]	4.7 [2.2, 7.9]	0.003
Daily use medication			
H_2_ blockers	2 (3)	1 (3)	0.25
Beta blockers	20 (33)	13 (37)	0.70
Steroids	3 (5)	0 (0)	0.70
Benzodiazepines	8 (13)	9 (26)	0.13
Statins	28 (47)	13 (37)	0.37

Data are presented as median [1st Interquartile range, 3rd Interquartile range] or cases (%). eGFR: estimated glomerular filtration rate.

**Table 2:** Intraoperative and ICU stay data of the patients with and without postoperative delirium.

	Delirium (−) *n* = 60	Delirium (+) *n* = 35	*p*-Value
Surgical procedure			
CABG using CPB	22	7	0.39
Valvular	22	15	
Arch replacement	9	7	
Combined	7	6	
Anesthetic data			
Anesthesia time (min)	451 [394, 498]	517 [382, 663]	0.05
CPB time (min)	150 [117, 202]	177 [128, 287]	0.12
Blood loss (mL)	1321 [984, 2021]	1782 [965, 3177]	0.57
ICU stay data			
Duration of ICU stay (h)	48.8 [43.8, 89.3]	91.9 [71.5, 158.1]	<0.001
Number of CAM-ICU evaluations	9 [6.75, 13]	15 [10.5, 26.5]	<0.001

Data are presented as a median [1st Interquartile range, 3rd Interquartile range]. ICU: intensive care unit, CABG: coronary artery bypass grafting, CPB: cardiopulmonary bypass, CAM: confusion assessment method.

**Table 3:** Odd ratios of low preoperative hand grip strength for postoperative delirium.

	Odds Ratio	95% Confidence Interval	*p*-Value
Model 1	4.23	1.69–10.5	0.002
Model 2	4.58	1.57–13.2	0.005

## Data Availability

The data that support the findings of this study are available from the corresponding author upon reasonable request.
